# Post-2015, why delay to seek healthcare? Perceptions and field experiences from TB healthcare providers in northern Malawi: a qualitative study

**DOI:** 10.1186/s40249-017-0279-1

**Published:** 2017-05-11

**Authors:** Nathan B. W. Chimbatata, Chang-Ming Zhou, Chikondi M. Chimbatata, Biao Xu

**Affiliations:** 1 School of Public Health, Fudan University, 138 Yi Xue Yuan Road, Xuhui District, Shanghai, 200032 China; 2 Key Laboratory of Public Health Safety (Ministry of Education), Shanghai, China; 3 Mzuzu University, Mzuzu, Malawi; 4 Mzuzu Central Hospital, Mzuzu, Malawi; 5 Department of Public Health Sciences (Global Health / IHCAR), Karolinska Institutet, Stockholm, Sweden

**Keywords:** Tuberculosis, Diagnosis delay, HIV stigma, Healthcare provider, Malawi

## Abstract

**Background:**

Malawi is a low-income country with high Tuberculosis (TB) burden. TB diagnosis delay and untimely initiation of treatment is still a major problem in Malawi which could increase the risk of tuberculosis transmission in the communities. This study investigated factors related to the diagnostic delay of tuberculosis from TB healthcare providers in the northern region of Malawi.

**Methods:**

Nine focus group discussions were conducted with 57 participants in total. The participants were healthcare cadres including district TB officers, clinical officers, TB nurses, laboratory technicians and Health Surveillance Assistants (HSAs). NVivo (11.0) software was used for data analysis.

**Results:**

The factors related to diagnostic delay were categorized into three themes: client factors, institutional factors and healthcare provider related factors. Client’s stigma and fear for HIV test, resource shortage within healthcare institutions and the healthcare workers’ poor attitude against potential patients were among the most influential factors behind the TB diagnostic delay.

**Conclusions:**

The TB control strategies should aim to reduce HIV stigma, improve resource supply and improve TB healthcare workers’ morale in order to achieve timely TB diagnosis.

**Electronic supplementary material:**

The online version of this article (doi:10.1186/s40249-017-0279-1) contains supplementary material, which is available to authorized users.

## Multilingual abstract

Please see Additional file 1 for translations of abstract into five official working languages of the United Nations.

## Background

Timely Tuberculosis (TB) detection and prompt treatment initiation are two crucial elements behind successful TB control. TB diagnosis delay leads to increased infectivity, disease burden and fatality. The consequences are severer in countries with high prevalence of TB/HIV co-infection that 30–50% HIV/AIDS cases die of TB [1]. In developing countries like Malawi, TB is still responsible for rising morbidity and mortality. In 2014, there were approximately 9.6 million TB incidents worldwide, whereas only 6 million cases were reported to the World Health Organization (WHO) [1]. In 2015, the global TB report had an estimation of 38 000 new TB cases in Malawi, whereas only 16 267 cases were notified. The gap between notification rate (97 per 100 000) and estimated incidence of TB (227 per 100 000) was remarkable. Also, the case detection rate of 43% was far below the Millennium Development Goal of 70% detection rate [1]. Such low case detection signifies a severe problem of diagnostic delay, either patient-related or health-system-related. Previous studies conducted in Malawi have reported that the diagnostic delay in TB varied from the median of 14 to 33.5 days [2, 3]. Low socio-economic status, old age, females, transportation difficulties, poor TB health service and lack of human resources could be reasons behind untimely TB diagnosis and treatment [4–9]. A systematic review in sub Saharan Africa revealed that delayed TB diagnosis could be due to long travel time to a healthcare provider or seeking help from a traditional healer [10]. Stigma and beliefs were also reported to be important factors in delaying healthcare seeking [11, 12]. Recent findings in Malawi also indicated a longer median health system delay of 59 days (IQR 26–108) as being due to clients’ initial visits to a lower level health facility [2]. Such delay was due to clients’ initial visits to less qualified health facilities [2].

Malawi is a low-income country shouldering a heavy disease burden of both TB and HIV/AIDS. Since early 1980s, TB control activities in Malawi have been coordinated by the National TB Control Program (NTP). TB health services are delivered at three planes: primary healthcare facilities to tertiary hospitals, government sponsorship and private profit as well as nonprofit organizations. At the community level, TB control activities are coordinated by HSAs [13]. Currently, the coverage of Directly Observed Treatment-Short Course (DOTS) strategy in Malawi is 100%. However, the high DOTS coverage does not come with a parallel improvement in case detection. The low TB case detection suggests delays in TB diagnosis and treatment [14].

Several studies have been done in Malawi looking at factors that influence client’s health care seeking from service recipients’ perspective [8, 9]. However, very few studies have viewed the issue from the healthcare provider’s perspective regarding their observation and experience. Under the full coverage of NTP in a population with very limited TB health knowledge, healthcare providers play a key role in providing prompt and proper TB diagnosis and treatment. Therefore, this study was conducted to explore field experience and perceptions of TB healthcare providers to study the risk factors which affect patient’s healthcare seeking and hinder timely TB diagnosis.

## Methods

This was a qualitative study employing focus group discussion.

### Study setting

The study was conducted in northern Malawi. Nine healthcare delivery facilities were included. The study site comprised one central hospital, five district hospitals, two mission hospitals and one health centre.

### Participants

Participants in this study were healthcare workers from different cadres, including district TB officers, clinical officers, TB nurses, laboratory technicians and HSAs.

### Data Collection

In 2016, nine focus group discussions (FGDs) were carried out across the nine health facility sites from March to June. A semi-thematised guide was used. The guide focused on obstacles in the care seeking process as well as experience on providing TB care. The participants were briefed about the study. Voluntary consent of participation was received from participants. All FGD sessions were recorded in audio and each session lasted averagely 40 to 55 min. These sessions were facilitated through active listening along with paraphrasing and probing upon requirements.

### Data Analysis

Nvivo 11.0 software was used for data analysis. Some data were analysed manually. FGD recordings were listened to repeatedly and then transcribed into documents by researchers. Codes were developed through repetitive reading of the transcripts. Then, researchers categorized data and developed major categories. The transcripts were shared with an independent researcher for separate analysis and coding. Any emerged minor differences were regularised through discussion.

## Results

### Characteristics of the study participants

In total, 57 healthcare providers, including 6 TB officers, 4 clinical officers, 3 TB nurses, 2 laboratory technicians and 42 HSAs participated in the FGDs. There were 23 females and 34 males. All participants have had TB caring experience of more than 2 years.

### Themes

The findings revealed that TB care had been going on smoothly regardless of the challenges it faced. The findings indicated that not all of the study sites had timely sputum smear microscope-based TB diagnosis. Reasons for diagnostic delays fell in the following three major themes.

#### Patient-related Factors

Several factors were related to the patients. These factors could delay care seeking and therefore hinder the timely diagnosis of TB.

- *Beliefs* Three participants (including 1 TB officer and 2 HSAs) stated that some patients did not seek healthcare timely due to their beliefs, either religious beliefs or spiritual ones.
*Actually most of the time, clients are not coming to the hospital in time as a result they spend most of the time at the prophets; saying that they will be healed if they have faith in Christ. So they do not report to hospital early.* (Male, TB Officer)
- *Stigma to HIV/AIDS* Eight participants (3 TB officers, 3 H.S.As and 2 Clinical Officers) mentioned that clients often suffered from stigma relating to HIV/AIDS. This statement was agreed by almost all other participating TB officers and HSAs. The stigma was considered to have stemmed from the fear of HIV amongst the people. Most patients feared that a TB diagnosis meant that they also had HIV. In other terms, they avoided seeking healthcare in fear of being diagnosed with HIV.
*There is a lot of stigma in the villages. So if one is found with TB they say aaah he has HIV. So they hide and do not come early to the hospital.* (Male, TB Officer)
  Some patients isolated themselves to avoid receiving an HIV notice. They often sought care in means that would not tell their HIV status, such as traditional healers. The stigma to HIV was very intensive.
*I just want to add, most of our TB patients, they are taking TB in relation to HIV. They may go to traditional healers thinking that they have been bewitched and it takes a longer period while there. When they are coming to us, their condition has already worsened as a result some do not survive.* (Male, Clinical officer)
- *Traditional healers* In most times, patients spent time seeking help from traditional healers, instead of a healthcare facility.
*Most clients when they cough they rush to traditional healers, so they spend a lot of time there and come here when they are very sick. Also some patients on ART when they see something growing on their body they do not realize that it could be TB and they go to traditional healers for traditional medicine while what they have are signs of TB.* (Female, senior HSA)
- *Lack of knowledge* Seven participants (including 2 Clinical officers and 5 HSAs), reported that the patients had very limited knowledge on TB and that they were not aware of TB symptoms. Several others nodded for it as well. In comparison, the community knew more of HIV than TB.
*Basically communities, they know much better information about HIV than TB. So our communities, they have just little knowledge about TB that’s why we are having these problems.* (Male, senior H.S.A)
- *Difficulties in Transportation* There were reports that it was difficult for patients to reach the nearest health facility due to distance, location and transportation difficulties. Many patients lived in mountain areas, some of them walked to the health facility from a remote location since there lacked well-built roads and vehicles that would reach these areas. Sometimes, patients were told to come back the next day to submit the morning and overnight sputum for diagnosis. These patients often failed to appear due to transportation difficulties.
*The geographical set up is not good, most areas are hilly.... You find that someone is from somewhere very far, poor road network, there is no car going that side so the patients have to walk to the health post.* (Male, TB Officer)

#### Institutional Factors

There were several shortfalls that affected hospitals and hindered timely diagnosis of TB. These factors included resource shortages, poor accessibility as well as poor capacity for TB diagnosis and weak community involvement.

- *Human and material resource shortage* Due to human resource shortage, patients had to be waiting in long queues for registration at the outpatient department. There were often queues for laboratory tests and other care services as well. Sometimes, patients decided to leave before being assisted. Some of them run away from queues and visited private practitioners instead, only to come back to public hospitals when their diseases aggravated.
*If you come here in the morning, there are long queues. So more time is spent on the queue than time for getting medical assistance. Sometimes they leave because they think they are wasting time, so often they go to their businesses before any help because they feel they are not very sick.* (Female, H.S.A)
  Six participants (involving 1 TB Officer, 2 Clinical Officers and 3 H.S.As) also observed largely compromised care delivery due to human resource shortage and the heavy working load. Hence, suspect TB patients were not adequately assessed.
*You can see we are having two, three clinicians working but with maybe over 300 patients so they do not take time to examine patients. As you know if the clinical inquiry is poor, the examination is poor; you end up having poor diagnosis and poor treatment.* (Male, Clinical officer).
  Lack of basic equipment and laboratory materials affected the effective delivery of TB diagnosis. In some hospitals, only one microscope was available and very few technicians were present to process samples, do the tests and respond to emergencies. There were also an insufficient number of professionals to take night shifts. Due to lack of laboratory technicians, the release of the test results was inevitably delayed.
*Sometimes a week would pass before the results are out.* (Female, TB nurse)
  There were also reports from participants indicating that the laboratory technicians feared to handle the sputum specimen at times, worried about the specimen’s infectivity. Therefore, the delay extended before the samples were processed in the laboratory.
- *Poor capacity for TB diagnosis* Three participants (including 1 Clinical Officer and 2 HSAs) stated that some facilities could not provide quality diagnosis for TB. These facilities lacked qualified staffs to run the smear microscope. Consequently, TB diagnosis was delayed. In fact, only hospitals at district or above levels were undoubtedly qualified for TB diagnosis.
*Just to add and make a comparison between TB and HIV. HIV can be diagnosed even at a rural level ……… not with TB. TB needs at least some expensive machines to diagnose a patient. Some health facilities do not have diagnostic sets for TB or they may have diagnostic sets but no personnel to use these and diagnose a patient but not with HIV, you just get determine and chase buffer you diagnose. So TB, there is need maybe if Government can have new knowledge and high technical expertise and introduce simple diagnostic TB sets and I think maybe we can reduce these other problems.* (Male, senior H.S.A)
- *Weak community involvement and health education* Two participants (1 TB Officer and 1 H.S.A) emphasized the lack of community involvement during the discussion as another crucial factor preventing timely TB diagnosis. This was agreed by almost all the HSAs. There were no community volunteers in most of the areas. Before, there were volunteers and sputum collection points. But they currently ran out of service due to lack of funding.
*Community involvement is not there, like volunteers, they are not there. In the past there was a special program specific for TB broadcasted at 6:00 am, but now it is not there. That could show that TB is gone. Even us health workers we think that TB is gone. But TB is there and it is being spread. Case finding is gone, but TB is ongoing and it is killing a lot of people and there are no awareness campaigns.* (Male, TB Officer)
  Sometimes there would be specific groups working with available incentives for certain focuses such as multidrug-resistant TB. These groups were helpful, although it could also repulse outside volunteers for the lack of incentive funding.

#### Healthcare provider related factors

The findings showed that knowledge, attitude and behaviours of a healthcare provider could also cause the diagnosis delay. The participants reported that sometimes the patients might have come to the hospitals in time but they were not assessed and examined adequately, delaying their TB diagnosis.

- *Under assessment* Findings from clinicians suggested that, although patients with TB symptoms should submit sputum for microscope exam, many were not told to do so. In many cases, care providers simply prescribed the patients with antibiotics because they did not relate the symptoms with TB. Some of the practitioners were not even updated in TB knowledge. Therefore, they were not reminded of the fact that coughing should be taken seriously. In the lab, some technicians didn’t know how to examine the sputum, did not know how the TB bacteria looked, and sometimes didn’t know how to use a microscope itself.
*When patients come for the first time with cough, they give them antibiotics, they come again, and they are given antibiotics as well just like that without telling these clients quickly to submit sputum.* (Female, HSA).
  And another participant stated:
*Talking about our laboratory personnel, some of them are not conversant with TB. For example, they might not know how to process the sputum, and they do not know how TB looks like under the microscopy.* (Male, Clinical Officer)
- *Unprofessional attitude from healthcare workers* Findings also revealed that the attitude of health workers affected the delivery of TB care. Sometimes, the healthcare workers were not very approachable at times. One participant shed light on the way patients were handled at times:
*Sometimes we shout at the patients and this shuns away the patients because they fear. For example, there was one patient from my catchment area; he was not fully examined because he was not an admitted patient. The patient returned home only to come later when he was critically ill. So us healthcare providers we should be dedicated.* (Female, H.S.A)
  One of the reasons for the bad attitude was because the healthcare workers were afraid they would be infected with TB. They were deeply concerned about the risk of contracting the disease from their patients. They were afraid of working in the TB wards. Some lab technicians were also afraid to handle and process the TB samples.

## Discussion

Timely diagnosis of TB is critical to improve TB detection and initiate appropriate treatment. Findings from this study manifest that factors which affected access to TB diagnosis in northern Malawi came mainly from three sources: the patient, the hospital and the healthcare provider.

### Patient factors

The study revealed that patients’ perceptions of TB, social and cultural factors had markedly influenced the patient’s healthcare seeking behaviours.

- *Lack of knowledge on TB* Since the TB awareness in the community was very low and generally wrong, it was unlikely for patients to relate themselves to TB when they were coughing or having more severe symptoms. Therefore, they were unlikely to consider hospitals for help in time. These findings are consistent with another study conducted in rural Malawi regarding poor knowledge on TB [15]. The same study reported that some participants believed that they were immune from TB since they were tested negative to HIV. This was due to the belief that TB was a disease for HIV patients. Such misconception on TB hinders patients from seeking TB care promptly. Findings of this study are also supported by studies in other settings such as China, where low education level, lack of TB knowledge and poor TB awareness were risk factors for delayed care seeking [16].
- *Stigma* A common finding in all FGDs was the stigma of HIV. It led to the fear of being discriminated and dehumanization treatment in society. In areas with high HIV prevalence like Malawi, more than half of the TB cases are HIV related. Simply because most of TB patients had HIV/AIDS, individuals thought if they were found with TB then they also had HIV. As a matter of fact, over 50% of TB patients are HIV co-infected in Malawi [1]. TB, in most cases, is the first manifestation of HIV. It is also the leading cause of death in HIV infected individuals [17]. The stigma was reported in two forms. The general community stigma, that highly believes of the co-existence of TB and HIV/AIDS; and the individual based stigma that a positive result of TB test would lead to an HIV test.
  Therefore, patients feared to seek hospital care to treat coughing in fear of being tested for HIV. These findings are consistent with study findings in other high HIV burden settings where TB patients in the communities faced HIV stigma because of the association between TB and HIV [18].
  The stigma could also be generated due to the fact that HIV is a sexually transmitted disease. In areas like Malawi, sex-related issues are not usually palatable for public discussion. Hence, people are reluctant to face their TB diagnosis because it has a strong link with HIV. This moral construction in most Sub Sahara African communities subjects the TB victims to condemnations that they could be infected with TB because they have been indulging themselves in immoral behaviour or breaking cultural norms [19]. TB has also been reported to be associated with immoral and disreputable behaviour in different Asian settings as well. A study in Nepal found that a woman with TB was suspected of being promiscuous in the past and that individuals with TB were regarded as having relations with different prostitutes in the past [20].
  The current study findings therefore highlight the need to promote TB and HIV awareness in the communities, to reduce the stigma and to give the positive messages that TB is preventable and curable if patients could reach TB care outlets timely.
- *Beliefs and Traditional healers* Patients’ belief system also played a major role in their care seeking behaviour. Many patients turned to different prophets at first, believing that these individuals had healing power. Some patients belonged to certain religious groups whose members were not allowed to seek medical help. These findings are consistent with those by Banerjee A, et al. [21] which revealed that the communities held different beliefs regarding the cause of TB, implying that the disease came due to ancestral spirits (*Mizimu*) or bewitchment (*Matsenga*). In a community with these beliefs, it is hard for an individual to think in the line of TB when they have a cough. It has also been noted in another study in Malawi that around 40% of patients visited traditional healers before they made their first visit to the formal healthcare facility [22]. It is believed that traditional medicine is effective in healing diseases caused by ancestral spirits and bewitchment. Similar situation was observed in Tanzania where TB patients associated their TB symptoms to sockery (kurogwa) or witchcraft (uchawi) [23]. Feedbacks from different settings supported the patient’s preference for traditional healers: that they were more accessible; they were recognized members of local communities who used the same language as of the patient’s; they had a high sense of social responsiveness and had compatible culture as the patient’s [24].
- *Low community involvement* Community involvement in TB care was found startlingly low in most of the catchment areas. Most frequently, TB attacks individuals with poor socioeconomic status and areas with limited access to information and health services [4]. Community involvement in TB care makes the individuals take responsibility over their own health. They seek care promptly when they experience a cough. In the meantime, community involvement is a key strategy used to stop TB, established by WHO in 2006. Therefore, low community involvement in TB care goes against the general regulations [25].

### Hospital factors

Hospitals often have a problematic care delivery system in Sub Sahara Africa [24]. There were institutional shortfalls regarding accessibility, diagnostic capacity and resources. Such insufficiency could hinder the timely diagnosis of tuberculosis.

- *Poor geographical accessibility* Difficulties in transportation are the main barriers barring timely healthcare seeking. In limited resource settings, traveling to the nearest healthcare facility is not easy to achieve [2, 25]. Healthcare facilities in Malawi are scattered. In addition, road network is incompetent especially in the northern region that is full of mountains. As a result, patients often had no other choices but to seek care from alternative sources that are usually informal. On the other hand, there lacked community sputum collection points and community TB volunteers in most of the catchment areas. Both situations lead to a break-off in the TB care chain in the remote communities. Thus, going to the nearest hospital outlet became one of the hardest decisions to make.
- *Shortage of resources* The shortage of human resource and required materials and equipment is a common issue in Malawi’s public hospitals [24]. The present study confirmed that healthcare facilities were stricken by resource shortages. Furthermore, most health centres had no capacity to diagnose TB. Lack of human resource became worse with heavy workloads. As a result, the patients sometimes had to wait in a long queue before they were helped. Very often, they left the hospital before it was their turn. The tedious waiting drove patients to care delivery outlets which, in most circumstances, had no capacity for making a TB diagnosis. Eventually, these patients would end up coming back to the public hospitals. Yet by that time, their conditions had often aggravated.

### Healthcare provider factors

In a setting where hospitals are scattered and are afflicted with persistent shortage of resources, healthcare providers experience challenges in healthcare delivery [26].

- *Knowledge gap on TB* It was found that some healthcare providers were not even qualified for delivering TB health services. The existing knowledge gap among healthcare providers was created by the lack of training and updates in TB care. Low involvement in TB-related affairs in general also disadvantages the care providers.
- *Negative attitude in the service* It appeared that some healthcare providers held a negative attitude towards TB patients. Sometimes, the patients were even shouted at by the workers. Similarly, a study in Ghana reported that healthcare workers had strong negative feelings against TB patients that they maltreated the patients and accused them for infecting the others [27]. Also, being designated to work at the TB section was perceived as a punishment among the workers [27]. In a similar vein, a study in Nepal found that TB suspect patients were stigmatized and discriminated against by health workers that sometimes a patient would leave the hospital before receiving any help [20]. It is these stigmatizing tendencies that would make clients not to reveal TB symptoms - or worse - not to seek medical care.

### Limitations

The study was conducted during the rain seasons. This made most areas nearly inaccessible. Therefore, most of the participants in the study were from district hospitals. The study was carried out among healthcare workers of different cadres and some cadres comprised more participants than others. This may have affected the diversity of the data obtained. Another limitation was that community volunteers did not participate in the study. This was mainly due to the volunteer mechanism on TB control in northern Malawi. The mechanism was basically gone. Thus it was difficult to locate any potential participants.

## Conclusions

In conclusion, TB patients in Malawi have not been timely diagnosed because of the stigma related to HIV/AIDS, low awareness of TB, poor community involvement and shortfalls in health system regarding diagnostic capacity, resources and perceptions of healthcare providers. If the TB diagnosis was delayed in the mentioned health facilities, there must be many patients with symptoms in the communities awaiting help. The fact was, what was observed in this study was only the visible top of an iceberg. The TB control strategies should aim at reducing HIV stigma, improving resource supplies and enhancing TB healthcare workers’ professionalism and morale in order to achieve timely TB diagnosis.

## Additional file

Additional file 1: Multilingual abstract in the five official working languages of the United Nations. (PDF 668 kb)

## Abbreviations

AIDS: Acquired Immune Deficiency Syndrome
CMB: China Medical Board
DOTS: Directly Observed Treatment Short course
FGD: Focus Group Discussion
HIV: Human immunodeficiency virus
HSA: Health Surveillance Assistant
NCST: National Commission of Science and Technology
NTP: National Tuberculosis Control Program
TB: Tuberculosis
WHO: The World Health Organization
