# Supplementary material for: Post-2015, why delay to seek healthcare? Perceptions and field experiences from TB healthcare providers in northern Malawi: a qualitative study
Source: Infect Dis Poverty. 2017 May 11;6:60. doi: 10.1186/s40249-017-0279-1 (PMC5426018; doi:10.1186/s40249-017-0279-1)
Supplement: Additional file 1: — Multilingual abstract in the five official working languages of the United Nations. (PDF 668 kb) [file 40249_2017_279_MOESM1_ESM.pdf]

Translation of the abstract into the five official working languages of the United Nations

ما بعد-2015، ما هي أسباب تأخر الحصول على الرعاية الصحية؟ انطباعات وتجارب ميدانية من مقدمي الرعاية الصحية لمرض السل في شمالي دولة مالاوي
دراسة نوعية.

ناثان بي. دبليو تشيمباتاتا، تشانغ-مينغ زو، تيكوندي إم. تشيمباتاتا بياوكسو

الخلاصة:
خلفية: تعتبر مالاوي دولة محدودة الدخل ذات معدلات عالية في الإصابة بمرض السل، حيث لا يزال تأخر تشخيص مرض السل وعدم الموائمة في البدء بعلاجه مشكلة مستعصية في مالاوي بما قد يزيد من خطر انتقال مرض السل بين السكان. تبحث هذه الدراسة في العوامل المتعلقة بتأخر تشخيص مرض السل من قبل مزودي الرعاية الصحية في الجزء الشمالي من مالاوي.
طرق البحث: أجرت الدراسة عدد تسع مناقشات في مجموعات تركيز تضمنت 57 مشاركا في مجملها. وقد تمثل المشاركون بالدراسة في كل من كوادر الرعاية الصحية بما فيهم الموظفين العاملين على المرض في المقاطعة، الموظفين السريريين، الممرضات العاملات على المرض، فنيي المعامل و مساعدي المراقة الصحية (H.S.A). وقد استخدم برنامج NVivo (11.0) في تحليل بيانات الدراسة.
النتائج: جرى تصنيف العوامل المتعلقة بتأخر تشخيص مرض السل إلى ثلاثة مواضيع كالتالي: العوامل الإكلينيكية، العوامل المؤسساتية، والعوامل المتعلقة بمقدمي الرعاية الصحية. حيث تمثلت أكثر العوامل تأثيرا وراء تأخر التشخيص في الوصمة المحتملة على المراجعين والخوف من اختبار مرض الإيدز، نقص الموارد في مؤسسات الرعاية الصحية، إضافة إلى الانطباعات السلبية التي يبديها العاملون في الرعاية الصحية تجاه المرضى المحتملين.
الخاتمة: يجب أن تهدف استراتيجيات التحكم في مرض السل إلى التقليل من الوصم بمرض الإيدز، وتحسين التزود بالموارد، إضافة إلى تحسين نظرة العاملين في الرعاية الصحية في هذا الإطار، وذلك للوصول إلى تشخيص مرض السل في وقته المناسب.

Translated from English version into Arabic by Norhan Mahmo, through

TRANSLATORS
WITHOUT BORDERS

后2015时代，缘何结核病就诊仍有延误？一项在马拉维北部开展的基于卫生服务提供者观点及经历的定性研究

Nathan B.W. Chimbatata, 周昌明, Chikondi M. Chimbatata, 徐飚

背景：马拉维是一个低收入国家，结核病负担高企。结核病患者的诊断延误和治疗延误迄今仍是一个严重问题，这一问题继而增加了结核病的社区传播风险。本研究旨在从卫生服务提供者的角度来探索马拉维北部地区结核病诊断延误的影响因素。
方法：研究采用焦点组访谈的方法，对9组共57名结核病医疗服务提供者进行了调查。受访者包括地区结核病项目官员、临床医生、护士、实验室技师以及卫生监测助理（HSA）。分析采用了内容分析法，并用NVivo (11.0) 对访谈内容进行资料处理。
结果：研究发现影响诊断延误的因素可归纳为三方面的主题，分别是患者、机构和卫生服务提供者相关因素。HIV污名及患者对HIV检测的恐惧、卫生服务机构资源的贫乏和医疗卫生人员对结核病疑似患者的不良态度均与结核病的诊断延误有关。

结论：结核病控制策略应着眼于减少对HIV的污名，改善卫生资源供给，提高结核病医疗卫生人员的道德观念，从而实现结核病的及时诊断。

Translated from English version into Chinese by Chang-Ming Zhou.

Après 2015, pourquoi tarder à se faire soigner ? Perceptions et expérience sur le terrain des prestataires de soins antituberculeux dans le nord du Malawi : étude qualitative

Nathan B.W. Chimbatata, Chang-Ming Zhou, Chikondi M. Chimbatata, Biao Xu

Résumé

Contexte : Le Malawi est un pays à faibles revenus où la tuberculose représente un fardeau important. Le retard du diagnostic de la tuberculose et de la mise en place du traitement y constituent encore un problème majeur, qui pourrait favoriser la transmission de la maladie au sein des communautés. Notre étude a examiné des facteurs liés au retard de diagnostic de la tuberculose rapportés par des prestataires de soins antituberculeux dans le nord du Malawi.

Méthodes : Neuf groupes de discussion ont été organisés avec un total de 57 participants, qui étaient des cadres de santé, notamment des responsables de la lutte antituberculeuse de district, des cadres cliniciens, des infirmiers et infirmières spécialisés dans la tuberculose, des techniciens de laboratoire des et assistants de surveillance sanitaire. Les données ont été analysées à l'aide du logiciel NVivo (11.0).

Observations : Les facteurs liés au retard de diagnostic ont été divisés en trois groupes thématiques : facteurs liés aux clients, aux institutions et aux prestataires de soins de santé. La stigmatisation qu'entraîne le dépistage du VIH et la crainte qu'en ont les clients, le manque de ressources dans les établissements de santé et la mauvaise attitude des professionnels de la santé vis-à-vis des patients potentiels comptent parmi les facteurs qui influent le plus sur le retard de diagnostic de la tuberculose.

Conclusion : Les stratégies de lutte contre la tuberculose devront chercher à réduire la stigmatisation attachée au VIH, à améliorer la disponibilité des ressources et à changer la mentalité des professionnels de la santé afin d'obtenir un diagnostic en temps utile de la tuberculose.

Translated from English version into French by Suzanne Assenat, through

TRANSLATORS
WITHOUT BORDERS

Почему после 2015 года происходят задержки в предоставлении медицинской помощи? Впечатления и опыт медицинских работников, борющихся с туберкулезом (ТБ) на севере Малави: качественное исследование

Натан Б. В. Чимбатата, Ченг-Минг Чжоу, Чиконди М. Чимбатата, Бяо Сю

Тезисы

Исходный контекст. Малави — бедная страна, несущая тяжкое бремя туберкулеза. Задержки при диагностике этого заболевания и несвоевременное начало лечения здесь по-прежнему являются серьезной проблемой. Указанные причины могут увеличить риск распространения ТБ среди населения. В этом исследовании рассматриваются факторы, из-за которых происходит задержка диагностики ТБ медицинскими работниками, борющимися с заболеванием на севере Малави.

Методы. Были проведены дискуссии среди 57 участников, разделенных на девять фокус-групп. В дискуссиях участвовали медицинские работники, в том числе участковые фтизиатры, служащие больниц, медицинские сестры (фтизиатрия), лаборанты и санитарные работники. Для анализа данных использовалось программное обеспечение NVivo (11.0).

Полученные данные. Факторы, влияющие на задержку диагностики, были разделены по трем темам: связанные с пациентами, учреждениями и медицинскими работниками. Страх пациента перед тестом на ВИЧ, дефицит материальных ресурсов в учреждениях здравоохранения и плохое отношение медицинских работников к потенциальным пациентам — вот одни из основных причин поздней диагностики ТБ.

Заключение. Стратегии борьбы с ТБ должны быть направлены на снижение страха перед тестом на ВИЧ, улучшение материального обеспечения учреждений здравоохранения и улучшение отношения работников, занимающихся борьбой с ТБ.

Translated from English version into Russian by Vadim Frolenko, through

TRANSLATORS WITHOUT BORDERS

Después del 2015, ¿Por qué retrasar la búsqueda de los cuidados sanitarios? Percepción y experiencia de campo de los profesionales de la salud para la TB en el norte de Malawi: un estudio cualitativo

Nathan B.W. Chimbatata, Chang-Ming Zhou, Chikondi M. Chimbatata, Biao Xu

Resumen

Antecedentes: Malawi es un país con bajos ingresos y con una carga elevada de Tuberculosis (TB). El retraso en diagnosticar la TB y la falta de un tiempo determinado para iniciar el tratamiento continúa siendo un gran problema en Malawi, que podría ver incrementado el riesgo de la transmisión de la tuberculosis en las comunidades. Este estudio investigó los factores relacionados con el retraso en el diagnóstico de la tuberculosis por parte de los profesionales de la saud en la región norte de Malawi.

Métodos: Se llevaron a cabo nueve grupos de discusión con un total de 57 participantes. Estos incluían cuadros sanitarios tales como oficiales TB de distrito, oficiales clínicos, enfermeras TB, técnicos de laboratorio y Ayudantes de Vigilancia Sanitaria (H.S.A por sus siglas en inglés). Se utilizó el software NVivo (11.0) para el análisis de datos.
Hallazgos: Los factores relacionados con el retraso en el diagnóstico se clasificaron en tres categorías: factores de personas, factores institucionales y factores relacionados con los profesionales sanitarios. Entre los factores que más influían en el retraso para diagnosticar la TB estaban la estigmatización de las personas y el miedo a efectuar el test del VIH, la escasez de recursos entre las instituciones sanitarias y la deficiente actitud del personal sanitario ante los pacientes potenciales.
Conclusión: Las estrategias para controlar la TB deberían enfocarse a reducir el estigma del VIH, mejorar el suministro de recursos y mejorar la moral del personal sanitario hacia la TB, para conseguir diagnosticar la TB a tiempo.

Translated from English version into Russian by Isabel Sanllehi, through

TRANSLATORS
WITHOUT BORDERS
